# A requirement for septins and the autophagy receptor p62 in the proliferation of intracellular *Shigella*

**DOI:** 10.1002/cm.21453

**Published:** 2018-09-10

**Authors:** Damián Lobato‐Márquez, Sina Krokowski, Andrea Sirianni, Gerald Larrouy‐Maumus, Serge Mostowy

**Affiliations:** ^1^ MRC Centre for Molecular Bacteriology and Infection, Department of Medicine Section of Microbiology, Imperial College London London United Kingdom; ^2^ Department of Immunology and Infection London School of Hygiene and Tropical Medicine, Keppel Street London United Kingdom; ^3^ MRC Centre for Molecular Bacteriology and Infection, Department of Life Sciences, Faculty of Natural Sciences Imperial College London London United Kingdom

**Keywords:** autophagy, cytoskeleton, metabolism, septin, *Shigella*

## Abstract

*Shigella flexneri*, a Gram‐negative enteroinvasive pathogen, causes inflammatory destruction of the human intestinal epithelium. During infection of epithelial cells, *Shigella* escape from the phagosome to the cytosol, where they reroute host cell glycolysis to obtain nutrients for proliferation. Septins, a poorly understood component of the cytoskeleton, can entrap cytosolic *Shigella* targeted to autophagy in cage‐like structures to restrict bacterial proliferation. Although bacterial entrapment by septin caging has been the subject of intense investigation, the role of septins and the autophagy machinery in the proliferation of noncaged *Shigella* is mostly unknown. Here, we found that intracellular *Shigella* fail to efficiently proliferate in SEPT2‐, SEPT7‐, or p62/SQSTM1‐depleted cells. Consistent with a failure to proliferate, single cell analysis of bacteria not entrapped in septin cages showed that the number of metabolically active *Shigella* in septin‐ or p62‐depleted cells is reduced. Targeted metabolomic analysis revealed that host cell glycolysis is dysregulated in septin‐depleted cells, suggesting a key role for septins in modulation of glycolysis. Together, these results suggest that septins and the autophagy machinery may regulate metabolic pathways that promote the proliferation of intracellular *Shigella* not entrapped in septin cages.

## 1 INTRODUCTION

*Shigella spp*. are Gram‐negative facultative enteroinvasive pathogens, closely related to *Escherichia coli*, that colonize the human intestinal epithelium and cause gastrointestinal illness known as shigellosis (Marteyn, Gazi, & Sansonetti, 2012; Kotloff et al., 2017). *Shigella* spp. are globally responsible for over 165 million illness episodes and 164 thousand deaths per annum (GBDDC, 2017; Lima, Havt, & Lima, 2015). Moreover, the World Health Organization (WHO) has highlighted *Shigella* as an urgent health threat due to widespread emergence of fluoroquinolone‐resistant strains (Chung & Baker, 2018; Harrington, 2015; WHO, 2017; Zhang et al., 2011). In addition to being an urgent health threat, *Shigella* is a paradigm for the investigation of cellular microbiology (Picking & Picking 2016). *Shigella spp*. possess a virulence plasmid encoding a type 3 secretion system (T3SS) that enables host cell invasion and intracellular proliferation (Mattock & Blocker 2017). Minutes after host cell internalization, *Shigella* escape from the phagocytic vacuole to the cytosol, where they proliferate (Ray, Marteyn, Sansonetti, & Tang, 2009). To support intracellular proliferation in epithelial cells, *Shigella* consume pyruvate derived from host cell glycolysis as a primary energy source (Kentner et al., 2014; Waligora et al., 2014). Although host cell metabolism is not significantly affected by the proliferation of intracellular *Shigella*, rapid catabolism of pyruvate into acetate is a hallmark of *Shigella* infection (Kentner et al., 2014). Interestingly, *Shigella* mutants unable to catabolize pyruvate into acetate can still proliferate intracellularly, albeit at a reduced rate (Kentner et al., 2014), and it has been suggested that alternative metabolic routes and/or carbon energy sources can be used by *Shigella* to sustain proliferation (Waligora et al., 2014).

To defend against *Shigella* invasion, host cells use a variety of mechanisms to restrict bacterial proliferation and dissemination including autophagy (Ogawa et al., 2005), guanylate‐binding proteins (GBPs) (Li et al., 2017; Piro et al., 2017; Wandel et al., 2017), and septin‐mediated cellular immunity (Mostowy et al., 2010; Sirianni et al., 2016). Septins are highly conserved GTP‐binding proteins that associate with actin filaments and cellular membranes (Mostowy & Cossart 2012; Spiliotis, 2010). The 13 human septins are classified into four homology groups (SEPT2, SEPT3, SEPT6, and SEPT7), and septins from different groups form hetero‐oligomers that assemble into nonpolar filaments. Septin filaments contain SEPT2 and SEPT6 family members, and are critically dependent on SEPT7 (Sirajuddin et al., 2007; Nakahira et al., 2010). Septins have key roles in numerous cellular processes, including cell division and host–pathogen interactions (Mostowy & Cossart, 2012; Torraca & Mostowy, 2016). Septins have also been shown to play important roles in cellular homeostasis, controlling store‐operated Ca^2+^ entry (SOCE) (Sharma et al., 2013), vesicle trafficking (Spiliotis, Hunt, Hu, Kinoshita, & Nelson, 2008) and mitochondrial fission (Pagliuso et al., 2016; Sirianni et al., 2016). Despite recent progress, a role for septins in host cell metabolism has not been investigated.

During *S. flexneri* infection, septins entrap actin‐polymerizing bacteria targeted to autophagy in cage‐like structures (Mostowy et al., 2010; Sirianni et al., 2016). Although bacterial entrapment by septin caging has been the subject of intense investigation, the role of septins and the autophagy machinery in the proliferation of noncaged *Shigella* is mostly unknown. In this study, we tested the role of septins (SEPT2, SEPT7) and the autophagy receptor p62/SQSTM1 in the proliferation of intracellular *Shigella* not entrapped in septin cages. Our data reveal a new role for septins and p62 in *Shigella* proliferation, and an unexpected role for septins in host cell glycolysis.

## 2 RESULTS AND DISCUSSION

### 2.1 A requirement for septins and p62 in the proliferation and metabolic activity of intracellular *Shigella* not entrapped in septin cages

**Figure 1:**
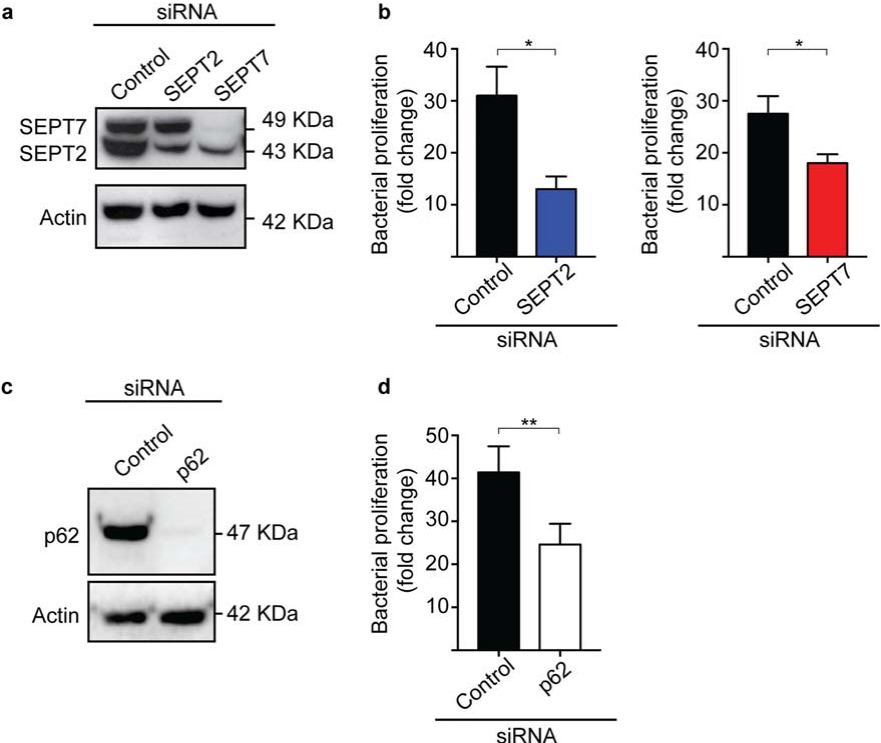
SEPT2, SEPT7 and p62 promote the proliferation of intracellular *Shigella*. (a) HeLa cells were treated with control, SEPT2, or SEPT7 siRNA sequences for 72 h. Whole‐cell lysates were immunoblotted for SEPT2 and SEPT7 to show the efficiency of depletion. Actin was used as a loading control. (b) siRNA‐treated cells were infected with *S. flexneri* str. M90T for 1 h 40 min or 4 h 40 min, then lysed and plated on LB‐agar plates. Graphs represent mean fold replication ratio 4 h 40 min/1 h 40 min ± SEM from four independent experiments. (c) HeLa cells were treated with control or p62 siRNA sequences for 72 h. Whole‐cell lysates were immunoblotted for p62 to show the efficiency of depletion. Actin was used as a loading control. (d) siRNA‐treated cells were infected with *S. flexneri* str. M90T as in (b) [Color figure can be viewed at wileyonlinelibrary.com]

To investigate a role for septins in the proliferation of intracellular *Shigella*, we performed gentamicin survival assays (Krokowski & Mostowy, 2016). HeLa cells were treated with small interfering RNA (siRNA) sequence specific for SEPT2 (Figure 1a; Supporting Information Figure S1), and infected with *S. flexneri* M90T for up to 4 h 40 min. Considering that septins regulate bacterial entry into host cells bacterial burden at 4 h 40 min postinfection was normalized to values at 1 h 40 min postinfection as previously described (Mostowy et al., 2009a,b). Unexpectedly, the depletion of SEPT2 (Figure 1a) resulted in significantly reduced (2.6 ± 0.5‐fold) bacterial proliferation, as compared to control cells (Figure 1b). To confirm this unexpected role for septins in *Shigella* proliferation, we infected HeLa cells treated with siRNA sequence specific for SEPT7 (Figure 1a). As previously shown (Estey et al., 2010), the depletion of SEPT7 also reduced levels of SEPT2 (Figure 1a; Supporting Information Figure S1). In agreement with results obtained for SEPT2‐depleted cells, *Shigella* proliferated significantly less (1.5 ± 0.1‐fold) in SEPT7‐depleted cells, as compared to control cells (Figure 1b). Septins mediate host cell division (Hartwell, 1971; Surka, Tsang, & Trimble, 2002), and may play a role in host cell viability (and therefore *Shigella* intracellular proliferation). To test this, we measured host cell viability in control‐, SEPT2‐, or SEPT7‐siRNA treated cells. We did not observe significant differences in viability between control and septin depleted cells (Supporting Information Figure S2a).

Factors controlling the proliferation of intracellular *Shigella* are not fully known. Previous work has shown that septins can modulate autophagy (Barve et al., 2018; Mostowy et al., 2010). Additionally, p62 has been demonstrated to facilitate *Salmonella* replication inside HeLa cells (Yu et al., 2014). Considering this, we tested the role of p62 in the proliferation of intracellular *Shigella*. We infected HeLa cells treated with siRNA sequence specific for p62 (Figure 1c). Consistent with septin–autophagy interactions, the depletion of p62 resulted in significantly reduced (1.7 ± 0.2‐fold) bacterial proliferation, as compared to control cells (Figure 1d). In agreement with results obtained using septin‐depleted cells, p62 depletion did not significantly affect host cell viability (Supporting Information Figure S2b). Together, these data suggest a requirement for septins and p62 in the proliferation of intracellular *Shigella*.

**Figure 2:**
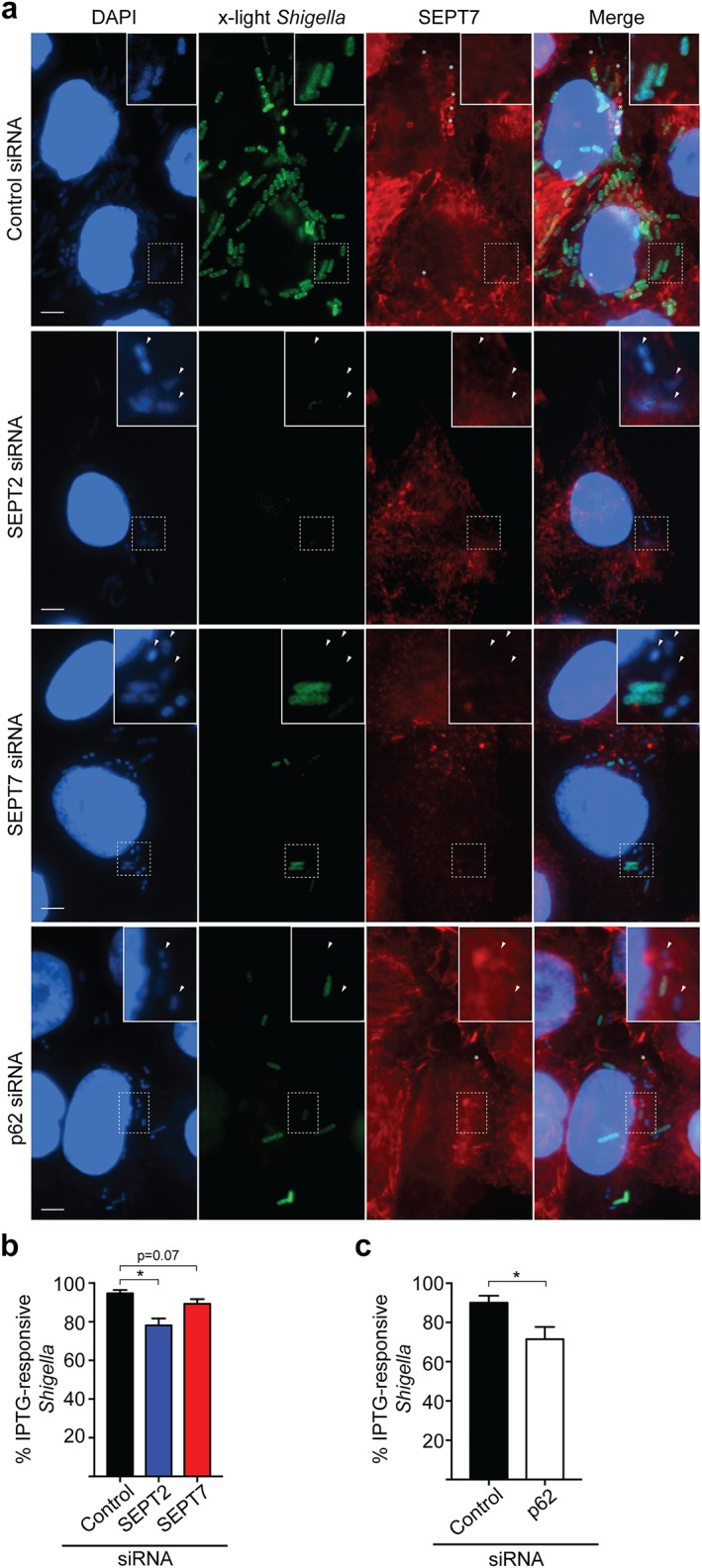
SEPT2, SEPT7, and p62 promote the metabolic activity of intracellular *Shigella* not entrapped in septin cages. (a) siRNA‐treated HeLa cells were infected with x‐light *S. flexneri* for 4 h 10 min, before being exposed to IPTG during 30 min prior to fixation. Samples were labeled for SEPT7 antibody to exclude septin caged bacteria from analysis. Arrowheads indicate metabolically inactive bacteria. *, septin cages. Scale bars = 5 μm. (b,c) HeLa cells were infected with x‐light *S. flexneri* as described in (a) and then the number of IPTG‐responsive intracellular bacteria was quantified in SEPT2‐, SEPT7‐ (b), or p62‐depleted cells (c). Graph represents mean % IPTG‐responsive bacteria ± SEM from four independent experiments. *p < .05 as analyzed by one‐way ANOVA (b) or Student's *t* test (c) [Color figure can be viewed at wileyonlinelibrary.com]

We reasoned that a reduction in the number of metabolically active intracellular bacteria may contribute to the decreased proliferation of *Shigella* in septin‐ or p62‐depleted cells. To test this, we used a *S. flexneri* strain (called ‘x‐light’) carrying a GFP‐encoding plasmid, where *gfp* expression is induced upon IPTG exposure (Schlosser‐Silverman et al., 2000; Sirianni et al., 2016). In this system, only metabolically active bacteria synthesize GFP upon IPTG exposure. Control, SEPT2‐, SEPT7‐, or p62‐depleted cells were infected with x‐light *S. flexneri* for 4 h 10 min, and IPTG was added for 30 min prior to fixation. After this, the percentage of bacteria responding to IPTG (and therefore metabolically active) was quantified (Figure 2a–c). Septin cages target bacteria to autophagy (Mostowy et al., 2010), so we labeled fixed cells with endogenous SEPT7 to exclude septin‐caged bacteria from analysis (Figure 2a). Consistent with *Shigella* being a facultative intracellular pathogen (Ray et al., 2009), 94.7 ± 1.7% of intracellular *Shigella* are metabolically active in control siRNA‐treated cells. In contrast, 78.1 ± 3.6, 89.3 ± 2.4, or 71.4 ± 6.3% of intracellular *Shigella* are responsive to IPTG in SEPT2‐, SEPT7‐, or p62‐depleted cells, respectively (Figure 2b,c). Collectively, these data suggest that septins and p62 support the metabolic activity of intracellular *Shigella* not entrapped in septin cages.

### 2.2 Septins control host cell glycolysis

**Figure 3:**
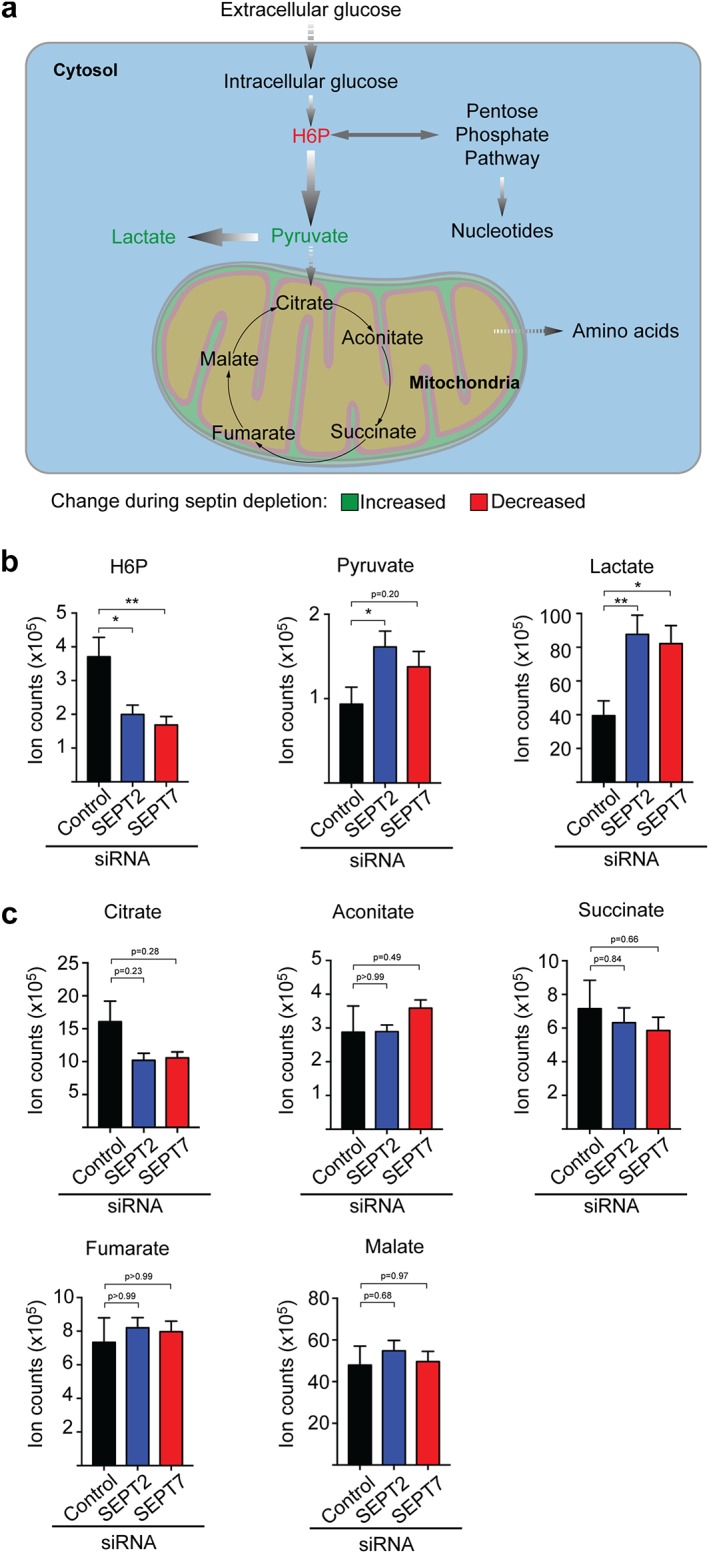
SEPT2 and SEPT7 regulate glycolysis in human epithelial cells. (a) Scheme of the primary metabolic routes in human epithelial cells. Arrows indicate the flux of each metabolic pathway. H6P, hexose‐6‐phosphate. (b,c) HeLa cells were treated with control, SEPT2, or SEPT7 siRNA sequences for 72 h. Cell lysates were prepared for LC–MS AMRT analysis. (b) Individual quantifications for indicated glycolytic metabolites, (c) individual quantifications for indicated TCA cycle metabolites. Graphs represent mean ± SEM of normalized ion counts from three independent experiments done in duplicate. *p < .05, **p < .001 as analyzed by one‐way ANOVA [Color figure can be viewed at wileyonlinelibrary.com]

Why is *Shigella* dependent on septins for intracellular proliferation? Considering that both the proliferation and metabolic activity of *Shigella* are reduced by septin depletion, we reasoned that host cell glycolysis (the primary energy source used by intracellular *Shigella* for proliferation) is also reduced. To test this, we used liquid chromatography–mass spectrometry accurate mass retention time (LC–MS AMRT) to analyze metabolites of the glycolysis pathway (Figure 3a) in the presence (control siRNA) or absence of septins (SEPT2 or SEPT7 siRNAs). Strikingly, LC–MS AMRT analysis showed that key intermediates of the glycolysis pathway are significantly dysregulated in septin‐depleted cells. The first intermediate of the glycolysis pathway, hexose‐6‐phosphate (H6P), is significantly decreased (2.0 ± 0.2 and 2.5 ± 0.5‐fold) in SEPT2‐ and SEPT7‐depleted cells, respectively, compared to control cells (Figure 3a,b). These results suggest that glycolysis may be impaired in septin‐depleted cells, and therefore glucose cannot be converted into H6P. Alternatively, glycolytic activity may be increased in septin‐depleted cells, and therefore H6P is consumed faster. To distinguish between these two possibilities, we tested the cellular levels of lactate (the final product of glucose fermentation) by LC–MS AMRT analysis. Consistent with a role for septins in suppression of glycolytic activity, we observed significantly more lactate (2.2 ± 0.3 and 2.1 ± 0.3‐fold) in SEPT2‐ and SEPT7‐depleted cells, respectively, as compared to control cells (Figure 3b).

To support intracellular proliferation in epithelial cells, *S. flexneri* consumes glycolysis‐derived pyruvate (Kentner et al., 2014). Surprisingly, we observed increased pyruvate levels (1.7 ± 0.2‐fold and 1.5 ± 0.2‐fold) in SEPT2‐ and SEPT7‐depleted cells, respectively, as compared to control cells (Figure 3b). These data suggest reduced *Shigella* proliferation in septin‐depleted cells is not due to lack of host‐derived pyruvate. Pyruvate can be converted into lactate in the cytosol, or be processed by the tricarboxylic acid (TCA) cycle in mitochondria (Figure 3a). Therefore, we reasoned that TCA cycle blockage may account for the accumulation of pyruvate and lactate observed in septin‐depleted cells. To test this, we analyzed TCA cycle metabolites by LC–MS AMRT analysis (Figure 3a,c). However, we failed to detect significant differences between control and SEPT2‐ or SEPT7‐depleted cells, for any of five TCA cycle metabolites tested (i.e., citrate, aconitate, succinate, fumarate, and malate) (Figure 3c). Decreased levels of H6P and increased levels of pyruvate and lactate in septin‐depleted cells may reflect altered protein levels of glycolytic enzymes. To test this, we measured the amount of four key enzymes of the glycolytic pathway, that is, hexokinase (HK), glyceraldehyde‐3‐phosphate dehydrogenase (GAPDH), pyruvate kinase (PK), and lactate dehydrogenase (LDH), in control‐, SEPT2‐, or SEPT7‐depleted cells by Western blot. We did not observe any change in the protein levels of HK, GAPDH, PK, or LDH upon septin depletion (Supporting information Figure S3).

Collectively, mass spectrometry analysis revealed that host cell glycolysis, crucial for the proliferation of intracellular *Shigella*, is dysregulated in septin‐depleted cells. Previous work has shown that septin recruitment to intracellular *Shigella* is dependent on p62 recruitment, and vice versa (Mostowy et al., 2010). Considering the established link between septins and p62, we hypothesized that p62 may modulate glycolysis similarly to septins. We employed LC–MS AMRT to analyze metabolites of the glycolytic pathway in control or p62‐depleted cells. In this case, we did not observe significant differences in levels of the glycolytic metabolites tested, indicating a specific role for septins in modulating glycolysis (Supporting Information Figure S4). These results demonstrate that, in the absence of septins or p62, bacterial proliferation is not compromised because of decreased pyruvate *per se*, and suggest that *Shigella* requires other metabolites dependent upon septins and/or p62 to support its efficient proliferation inside host cells. It is widely recognized that intracellular bacterial pathogens require a vast repertoire of nutrients to sustain proliferation (Abu Kwaik & Bumann 2015; Eisenreich, Dandekar, Heesemann, & Goebel, 2010). The precise role of septins and p62 in host cell metabolism and bacterial proliferation awaits investigation.

Previous work from our lab has shown that septins entrap cytosolic *Shigella* in cage‐like structures and target bacteria to autophagy (Mostowy et al., 2010; Sirianni et al., 2016). Here, we show that septins are required for the intracellular proliferation of noncaged *Shigella*. This suggests a dual role for septins during *Shigella* infection: septins can act as a cellular defense mechanism to restrict bacterial infection by forming cages to entrap bacteria targeted to autophagy, but also control metabolic pathways required for the proliferation of intracellular *Shigella* not entrapped in septin cages. While autophagy is widely recognized as an anti‐bacterial defense mechanism, there is increasing evidence that some intracellular pathogens, for example *Salmonella* Typhimurium, *Brucella abortus*, and *Legionella pneumophila*, have mechanisms to exploit the autophagy machinery for intracellular proliferation (Choy et al., 2012; Starr et al., 2012; Yu et al., 2014). As shown here for *Shigella*, the intracellular proliferation of the eukaryotic parasite *Toxoplasma gondii* is decreased when autophagy is inhibited (Pernas, Bean, Boothroyd, & Scorrano, 2018). Interestingly, *T. gondii* induces the autophagy of lipid droplets (i.e., lipophagy) to obtain fatty acids from the host cell required for its proliferation. Similarly, autophagy‐derived fatty acids may support the proliferation of intracellular *Shigella*, as also suggested in the case of *Salmonella* (Yu et al., 2014). Future work will be required to investigate the underlying requirement for septins and p62 in the metabolism and proliferation of intracellular *Shigella*.

What is the role of the cytoskeleton in host cell glycolysis? The glycolytic pathway is viewed to constitute a ‘metabolon’, a multienzymatic complex that enables metabolite channeling (Moraes & Reithmeier, 2012; Ovadi & Saks, 2004), and components of the cytoskeleton are recognized to interact with the glycolysis metabolon (Araiza‐Olivera et al., 2013; Waingeh et al., 2006). In this case, glycolytic enzymes can bind to actin filaments or microtubules, and these interactions modulate metabolon stability and metabolite channeling (Araiza‐Olivera et al., 2013; Lehotzky, Telegdi, Liliom, & Ovadi, 1993; Mejean, Pons, Benyamin, & Roustan, 1989; Vertessy, Orosz, Kovacs, & Ovadi, 1997; Waingeh et al., 2006). For example, work has shown that actin filaments bind glyceraldehyde‐3‐phosphate dehydrogenase (GAPDH), increasing its enzymatic activity (Poglazov & Livanova, 1986). What is the role of septins in glycolysis? Septins form molecular scaffolds and diffusion barriers, interacting with actin for cellular compartmentalization (Mostowy & Cossart, 2012; Saarikangas & Barral, 2011). Considering the roles for actin in glycolysis (Araiza‐Olivera et al., 2013; Mejean et al., 1989; Waingeh et al., 2006), it is tempting to speculate that septins can also modulate glycolysis. In agreement with this, our results show that septin depletion significantly increases glycolytic activity (Figure 3b) without affecting the protein levels of glycolytic enzymes (Supporting Information Figure 3). Here, septins may inhibit glycolytic enzymes, such as hexokinase (that mediates the phosphorylation of glucose into H6P), and therefore inhibit glycolysis. However, in the absence of septins, such enzymes would be free to interact with other components of the pathway, enabling rapid glucose catabolism. In support of this, recent work has shown that actin filaments can regulate glycolysis by inhibiting the glycolytic enzyme aldolase (Hu et al., 2016). In a separate study using human adipocytes, work has shown that SEPT11 interacts with Caveolin1 and the fatty acid chaperone FABP5 to control lipid traffic and metabolism (Moreno‐Castellanos et al., 2017). Together, a collective picture is emerging that cytoskeletal components, including septins, have crucial roles in the regulation of host cell metabolism.

Based on the results discovered here, we conclude that both septins and p62 are required for the proliferation of intracellular *Shigella*. We also show that septins can modulate host cell glycolysis. In this study, we use the human epithelial cell line HeLa as our infection model. HeLa cells, as other immortalized (tumor) cell lines, catabolize glucose via the Embden–Meyerhof–Parnas pathway or via aerobic glycolysis (Warburg effect) (Eisenreich, Dandekar, Heesemann, & Goebel, 2013; Kentner et al., 2014; Warburg, 1956). Other cell types, such as primary colonocytes, can present a different metabolism in which the TCA cycle is primarily used for energy production (Donohoe et al., 2012; Zhang, Wu, Chapkin, & Lupton, 1998). Considering this, it would be interesting to see how septins influence glycolysis and *Shigella* proliferation in human colonocytes, the primary cells targeted by *Shigella* during infection in humans. In the future, a complete understanding of how host cell metabolism is regulated by autophagy and the cytoskeleton can help develop novel approaches to combat infection.

## 3 EXPERIMENTAL PROCEDURES

### 3.1 Bacterial strains and plasmids

*Shigella* strains were grown in trypticase soy broth (TCS) agar containing 0.01% (w/v) congo red to select for red colonies, indicative of a functional T3SS. TCS liquid cultures were inoculated with Individual red colonies of *S. flexneri* M90T or x‐light (producing GFP upon addition of Isopropyl β‐d‐1‐thiogalactopyranoside, IPTG) and were grown overnight at 37°C with shaking. The following day, bacterial cultures were diluted in fresh prewarmed TCS (1:50 v/v), and cultured until an optical density (OD_600nm_) of 0.6. To grow x‐light *S. flexneri*, TCS was supplemented with 100 μg/mL of carbenicillin.

### 3.2 Cell cultures

HeLa (ATCC CCL‐2) cells were grown at 37°C and 5% CO_2_ in Dulbecco's Modified Eagle Medium (DMEM, GIBCO) supplemented with 10% fetal bovine serum (Sigma‐Aldrich).

### 3.3 Transfection, molecular probes, pharmacological inhibition

HeLa cells (7 × 10^4^) were plated in 6‐well plates (Thermo Scientific) for 16 h and then transfected with selected siRNAs as previously described (Mostowy et al., 2010; Sirianni et al., 2016). siRNA transfection was performed in DMEM with oligofectamine (Invitrogen) according to the manufacturer's instructions. Cells were tested 72 h after siRNA transfection. Control siRNA (ID#AM4635) and predesigned siRNA for SEPT2 (ID#14709), SEPT7 (ID#s2743) or p62 (ID#s16962) were all from Ambion.

### 3.4 Antibodies and Western blotting

Rabbit antibodies used were anti‐SEPT7 (ID#18991, IBL), anti‐Actin (ID#A2066, Sigma), anti‐Hexokinase‐II (ID#ab209847), anti‐Pyruvate kinase (ID#ab116271, abcam) and anti‐Lactate dehydrogenase (ID#ab52488, abcam). Anti‐SEPT2 (ID#60075‐1‐Ig, Proteintech Europe), anti‐GAPDH (ID#ab8245, abcam) and anti‐p62 (ID#610832, BD Biosciences) are mouse antibodies. Secondary antibodies used were goat anti‐mouse (ID#P0260, Dako) or anti‐rabbit (ID#P0448, Dako) antibodies, both horseradish peroxidase‐conjugated. Actin was used as a loading control. All antibodies were diluted in blocking solution (75 mM Tris‐HCl pH 8.8, 150 mM NaCl, 0.1% Tween20) supplemented with 3% fatty acid‐free milk.

For immunoblotting, siRNA transfected cells were washed 2× in PBS pH 7.4. Then to obtain total cellular extracts cells were lysed in Laemmli buffer (Laemmli, 1970) and incubated at 95°C for 10 min. Proteins were resolved in 8, 10, or 12% SDS–polyacrylamide gels and transferred to polyvinylidene difluoride membranes (PVDF, #IPVH00010, MerckMillipore).

### 3.5 Gentamicin survival assays

HeLa cells were seeded in 6‐well plates (Thermo Scientific) and treated with siRNAs as described above. Cell cultures were infected with *S. flexneri* at a multiplicity of infection (MOI, bacteria: cell) of 100:1. Bacteria and cells were centrifuged at 110×*g* for 10 min at room temperature, and then placed at 37°C and 5% CO_2_ for 30 min. Cell cultures were washed 2× with phosphate buffered saline (PBS) pH 7.4 and incubated with fresh DMEM containing 50 µg/mL gentamicin for 1 or 4 h. For gentamicin survival assays, 1 and 4 h‐infected cells were washed 3× with PBS pH 7.4 and lysed 5 min with 0.1% Triton X‐100 (in PBS) at room temperature. Cell lysates were serially diluted, plated on lysogeny broth (LB) plates and incubated at 37°C. Bacterial replication was quantified as the ratio of the number of colony forming units at 4 h 40 min relative to 1 h 40 min.

### 3.6 Quantification of metabolically active bacteria (x‐light *Shigella*)

For microscopy experiments involving x‐light *S. flexneri*, HeLa cells were seeded on glass coverslips in 6‐well plates and treated with siRNAs as described above. After 72 h of siRNA treatment cell cultures were infected with x‐light *S. flexneri* (MOI of 100:1, bacteria:cell) for 4 h 10 min‐infected and then treated with 0.1 mM IPTG for 30 min prior to fixation. Cells were washed 2× with PBS pH 7.4 and fixed 15 min in 4% paraformaldehyde at room temperature. Cells were washed 2× with PBS pH 7.4 and incubated with 50 mM ammonium chloride for 10 min. Cells were subsequently washed 2× PBS pH 7.4 and permeabilized 5 min with 0.1% Triton X‐100 (in PBS). Cells were then washed 3× in PBS and incubated with primary anti‐SEPT7 antibody diluted in PBS supplemented with 0.1% Triton X‐100 and 1% bovine serum albumin (#A2058, Sigma‐Aldrich). Secondary antibody and Hoechst incubations were performed in PBS supplemented with 0.1% Triton X‐100. Samples were preserved with aqua polymount mounting medium (ID#18606, Polyscience).

To quantify IPTG‐responsive bacteria, images were processed by ImageJ software. Before quantifying GFP positive bacteria brightness and contrast were adjusted for all images to remove noise signal from GFP channel, so only bacteria but not host cells could be seen. The total number of bacteria were counted using Hoechst stain. Then, GFP‐negative bacteria were quantified.

Microscopy images were acquired on fluorescence‐inverted microscope AxioObserver Z1 (Carl Zeiss MicroImaging) driven by ZEN software 2.0.

### 3.7 Quantification of dead cells

Cells were seeded in 6‐well plates and treated with siRNAs as described above. After 72 h cells were washed 2× with PBS pH 7.4 and incubated with 200 μL of trypsin for 5 min at 37°C and 5% CO_2_. Then, cells were suspended with 300 μL of DMEM. Cells were diluted 1:2 in trypan blue and the number of dead cells was measured by trypan blue dye exclusion using a hemocytometer; dead cells become blue in the presence of the dye.

### 3.8 Targeted metabolite analysis

HeLa cells were grown in 6‐well plates as described above. Cells were washed 2× in cold PBS pH 7.4. 1 × 10^6^–10^7^ cells were scraped and resuspended in 1 mL of lysis solution (acetonitrile/methanol/water 40:40:20 v/v/v) and transferred to 2 mL microtubes (#72.693.005, Starstedt). After 6–10 times mixing, cell lysate was transferred into a fresh Eppendorf tube and spun down 10 s at 17,000×*g*. The cleared supernatant was filtered into 0.2 μm Spin‐X column (Costar 8161). The flow‐through‐containing metabolomes extract was diluted 1:1 into acetonitrile containing 0.2% acetic acid prior to injection. Aqueous normal phase liquid chromatography was performed using an Agilent 1290 Infinity II LC system equipped with binary pump, temperature‐controlled autosampler (set at 4°C) and temperature‐controlled column compartment (set at 25°C), containing a Cogent Diamond Hydride Type C silica column (150 mm × 2.1 mm; dead volume 315 µL). A flow rate of 0.4 mL/min was used. Elution of polar metabolites was carried out using solvent A (0.2% acetic acid in deionized water (resistivity ∼18 MΩ cm), and solvent B (acetonitrile and 0.2% acetic acid). Mass spectrometry was carried out using an Agilent Accurate Mass 6545 QTOF apparatus. Nozzle Voltage and fragmentor voltages were set at 2,000 and 100 V, respectively. The nebulizer pressure was set at 50 psig and the nitrogen drying gas flow rate was set at 5 L/min. The drying gas temperature was maintained at 300°C. Data were collected in the centroid mode in the 4 GHz (extended dynamic range) mode. Ion counts were normalized to the amount of biomass of individual samples determined by the residual protein content of metabolite extracts using the BCA assays kit (Thermo^®^).

### 3.9 Statistics

Statistical analyses were performed using GraphPad Prism (v7, La Jolla, USA). Data are presented as mean ± standard error of the mean (SEM) from at least three independent experiments per treatment. One‐way ANOVA and Student's *t* test were used to compare values, with *p* < .05 considered as significant.

## Supporting information


**Supporting Figure S1. Depletion of SEPT2 does not affect levels of SEPT7 in human epithelial cells**. HeLa cells were treated with control, SEPT2 or SEPT7 siRNA sequences for 72 h. Whole‐cell lysates were immunoblotted for SEPT2 and SEPT7 to compare the protein levels of each septin. Actin was used as a loading control. Four independent experiments are shown here.

**Supporting Figure S2. Depletion of SEPT2, SEPT7 or p62 does affect viability of human epithelial cells**. HeLa cells were treated with control, SEPT2, SEPT7 (a) or p62 (b) siRNA sequences for 72 h. Then cells were stained with trypan blue and the number of dead cells quantified. Graphs represent mean percentage of dead cells ± SEM from at least four independent experiments. Data were analyzed by on‐way ANOVA (a) o Student's t‐tes (b).

**Supporting Figure S3. Depletion of SEPT2 or SEPT7 does not affect protein levels of glycolytic enzymes in human epithelial cells**. HeLa cells were treated with control, SEPT2 or SEPT7 siRNA sequences for 72 h. Whole‐cell lysates were immunoblotted for hexokinase‐II, glyceraldehyde‐3‐dehydrogenase, pyruvate kinase or lactate dehydrogenase. Actin was used as a loading control. Graphs represent mean fold change ± SEM of protein levels normalized to control‐siRNA treated cells and the loading control. Measurements come from at least four independent experiments performed in triplicate, and were analyzed by one‐way ANOVA.

**Supporting Figure S4. p62 does not modulate glycolysis in human epithelial cells**. HeLa cells were treated with control or p62 siRNA sequences for 72 h. Cell lysates were prepared for LC‐MS AMRT analysis. Individual quantifications for glycolytic metabolites H6P, pyruvate and lactate are shown. Graphs represent mean ± SEM of normalized ion counts from three independent experiments performed in duplicate.
