# Supplementary figures and images for: A requirement for septins and the autophagy receptor p62 in the proliferation of intracellular Shigella
Source: Cytoskeleton (Hoboken). 2018 Sep 10;76(1):163–72. doi: 10.1002/cm.21453 (PMC6519264; doi:10.1002/cm.21453)

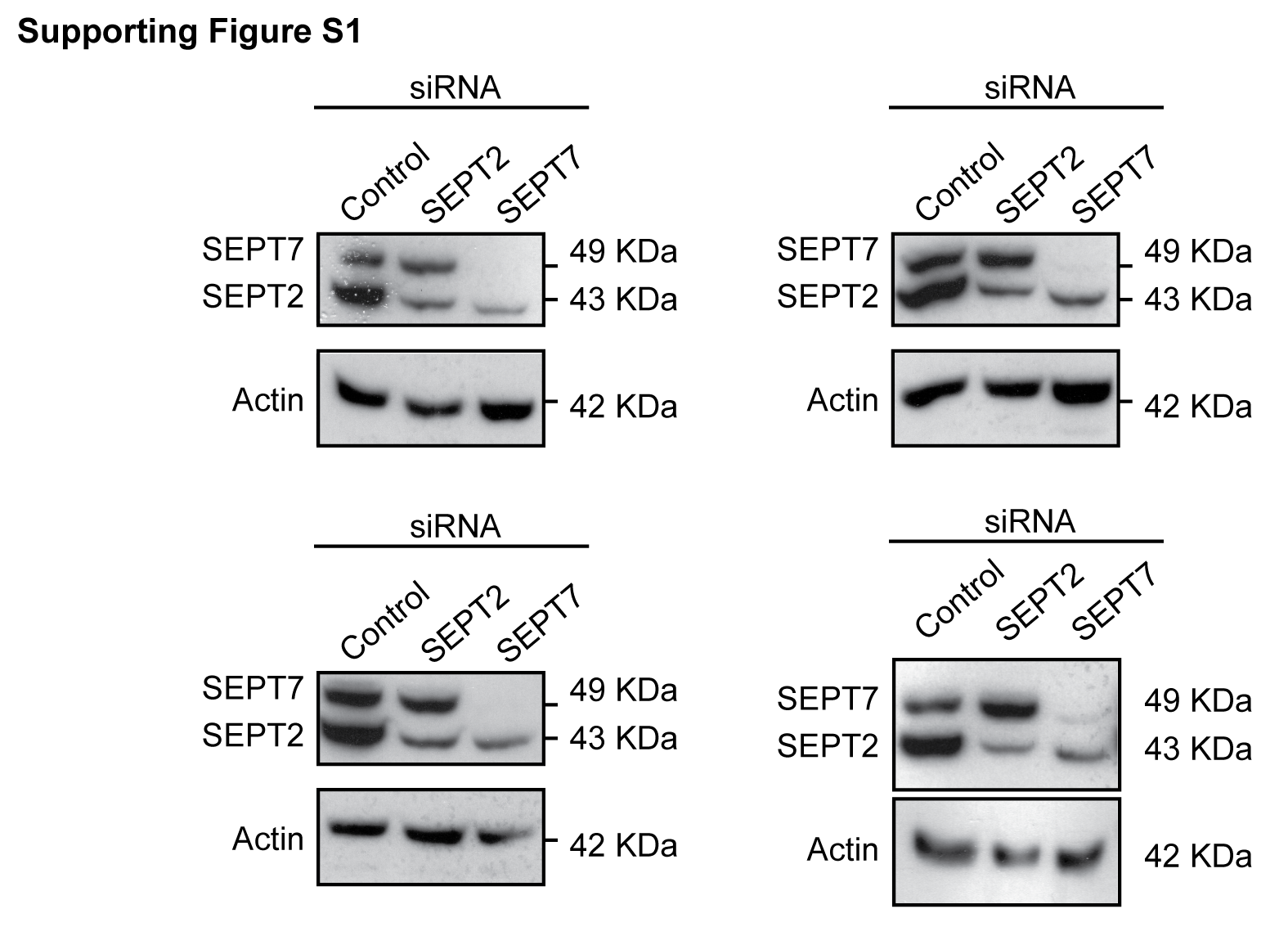

Supplement: Supplementary file 1 — Supporting Figure S1. Depletion of SEPT2 does not affect levels of SEPT7 in human epithelial cells. HeLa cells were treated with control, SEPT2 or SEPT7 siRNA sequences for 72 h. Whole‐cell lysates were immunoblotted for SEPT2 and SEPT7 to compare the protein levels of each septin. Actin was used as a loading control. Four independent experiments are shown here. [file CM-76-163-s001.tif]

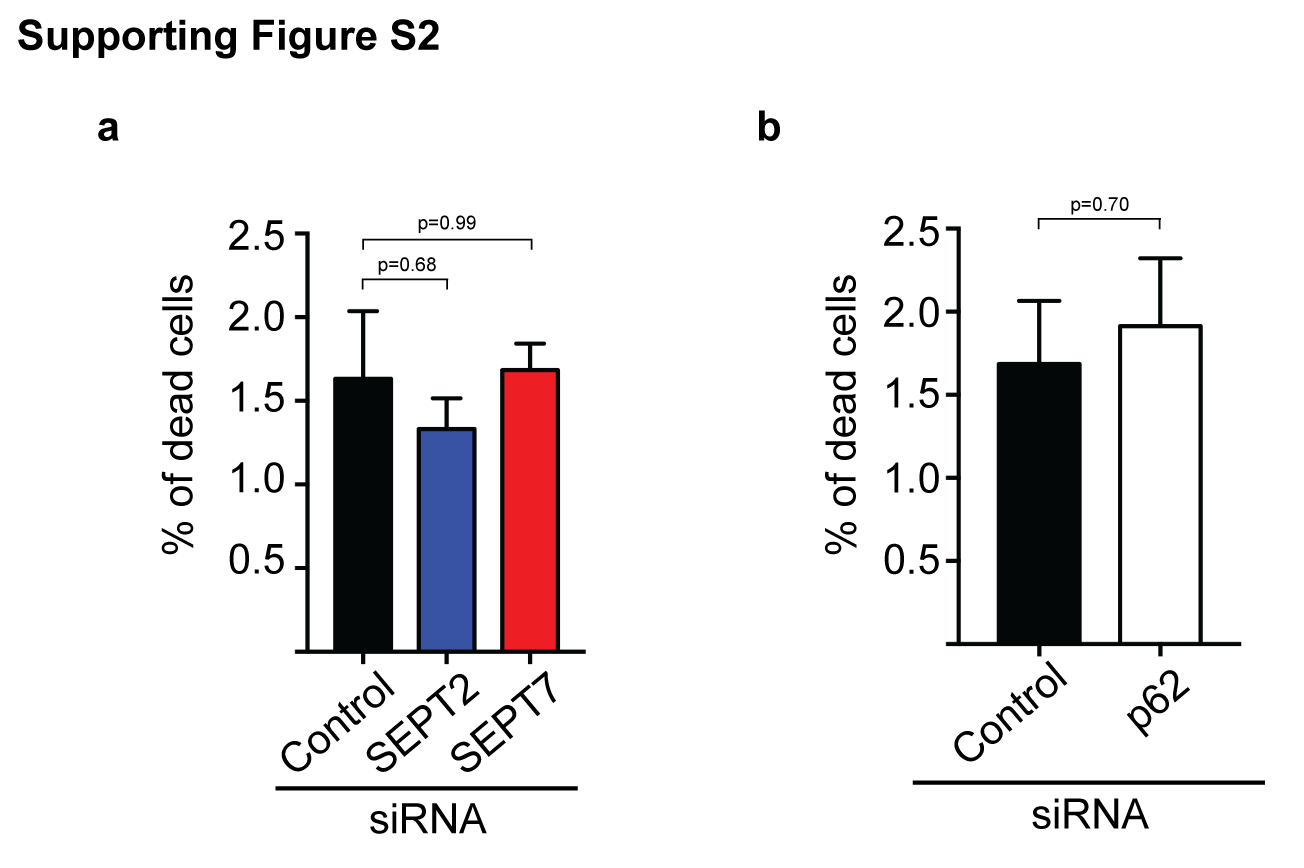

Supplement: Supplementary file 2 — Supporting Figure S2. Depletion of SEPT2, SEPT7 or p62 does affect viability of human epithelial cells. HeLa cells were treated with control, SEPT2, SEPT7 (a) or p62 (b) siRNA sequences for 72 h. Then cells were stained with trypan blue and the number of dead cells quantified. Graphs represent mean percentage of dead cells ± SEM from at least four independent experiments. Data were analyzed by on‐way ANOVA (a) o Student's t‐tes (b). [file CM-76-163-s002.tif]

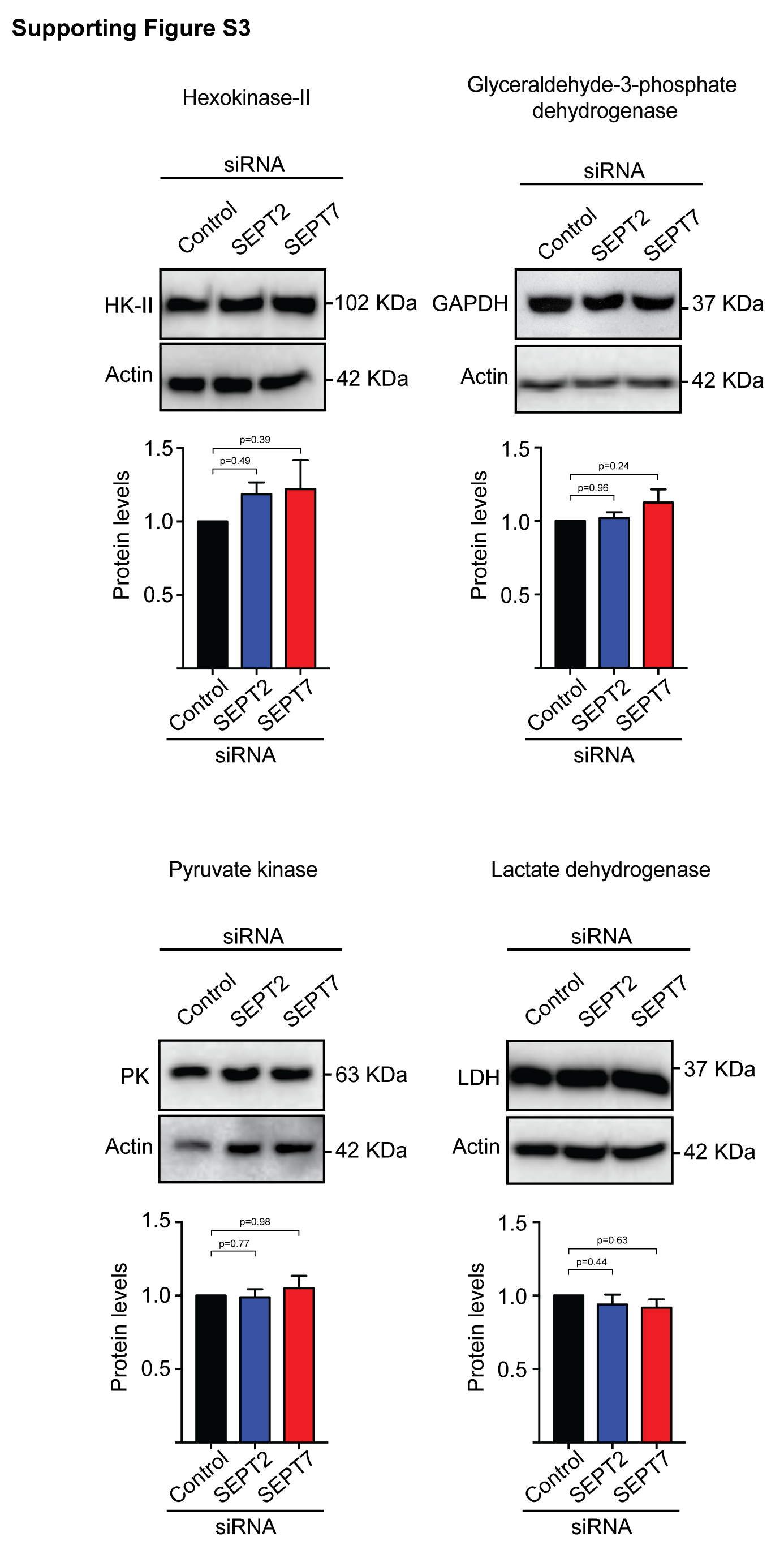

Supplement: Supplementary file 3 — Supporting Figure S3. Depletion of SEPT2 or SEPT7 does not affect protein levels of glycolytic enzymes in human epithelial cells. HeLa cells were treated with control, SEPT2 or SEPT7 siRNA sequences for 72 h. Whole‐cell lysates were immunoblotted for hexokinase‐II, glyceraldehyde‐3‐dehydrogenase, pyruvate kinase or lactate dehydrogenase. Actin was used as a loading control. Graphs represent mean fold change ± SEM of protein levels normalized to control‐siRNA treated cells and the loading control. Measurements come from at least four independent experiments performed in triplicate, and were analyzed by one‐way ANOVA. [file CM-76-163-s003.tif]

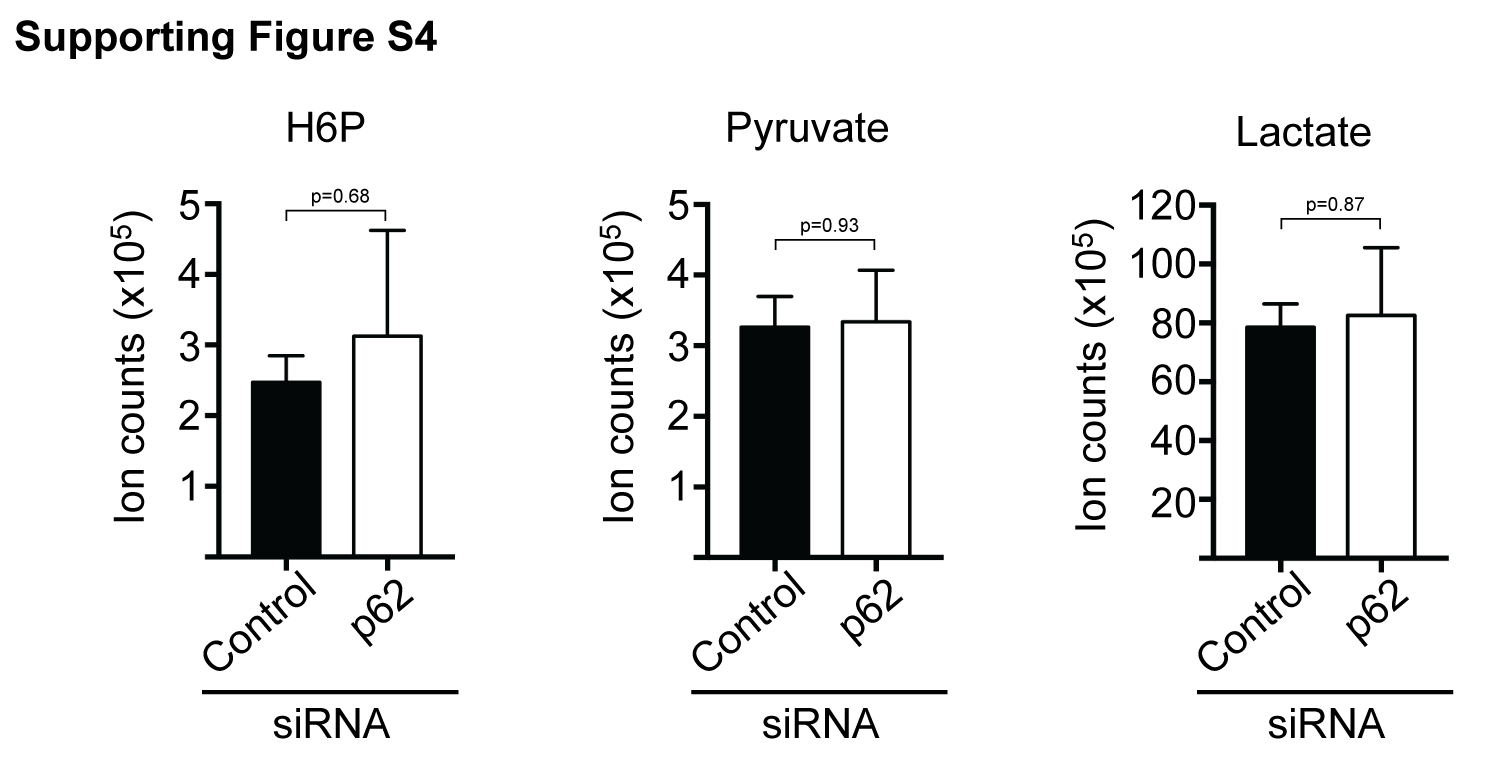

Supplement: Supplementary file 4 — Supporting Figure S4. p62 does not modulate glycolysis in human epithelial cells. HeLa cells were treated with control or p62 siRNA sequences for 72 h. Cell lysates were prepared for LC‐MS AMRT analysis. Individual quantifications for glycolytic metabolites H6P, pyruvate and lactate are shown. Graphs represent mean ± SEM of normalized ion counts from three independent experiments performed in duplicate. [file CM-76-163-s004.tif]
